# Epidemiology of dengue virus infections in Nepal, 2006–2019

**DOI:** 10.1186/s40249-021-00837-0

**Published:** 2021-04-15

**Authors:** Komal Raj Rijal, Bipin Adhikari, Bindu Ghimire, Binod Dhungel, Uttam Raj Pyakurel, Prakash Shah, Anup Bastola, Binod Lekhak, Megha Raj Banjara, Basu Dev Pandey, Daniel M. Parker, Prakash Ghimire

**Affiliations:** 1 Central Department of Microbiology, Tribhuvan University, Kirtipur, Kathmandu Nepal; 2 Centre for Tropical Medicine and Global Health, Nuffield Department of Medicine, University of Oxford, Oxford, UK; 3 Mahidol Oxford Tropical Medicine Research Unit, Bangkok, Thailand; 4 Epidemiology and Diseases Control Division (EDCD), Department of Health Service, Ministry of Health and Population, Teku, Kathmandu, Nepal; 5 Sukraraj Tropical and Infectious Disease Hospital Teku, Kathmandu, Nepal; 6 University of California, Irvine, CA USA

**Keywords:** Dengue, DENV, *Aedes aegypti*, Outbreak, Nepal, Spatial epidemiology

## Abstract

**Background:**

Dengue is one of the newest emerging diseases in Nepal with increasing burden and geographic spread over the years. The main objective of this study was to explore the epidemiological patterns of dengue since its first outbreak (2006) to 2019 in Nepal.

**Methods:**

This study is a retrospective analysis that covers the last 14 years (2006–2019) of reported dengue cases from Epidemiology Diseases Control Division (EDCD), Ministry of Health and Population, Government of Nepal*.* Reported cases were plotted over time and maps of reported case incidence were generated (from 2016 through 2019). An ecological analysis of environmental predictors of case incidence was conducted using negative binomial regression.

**Results:**

While endemic dengue has been reported in Nepal since 2006, the case load has increased over time and in 2019 a total of 17 992 dengue cases were reported from 68 districts (from all seven provinces). Compared to the case incidence in 2016, incidence was approximately five times higher in 2018 [incidence rate ratio (IRR): 4.8; 95% confidence interval (*CI*) 1.5–15.3] and over 140 times higher in 2019 (IRR: 141.6; 95% *CI* 45.8–438.4). A one standard deviation increase in elevation was associated with a 90% decrease in reported case incidence (IRR: 0.10; 95% *CI* 0.01–0.20). However, the association between elevation and reported cases varied across the years. In 2018 there was a cluster of cases reported from high elevation Kaski District of Gandaki Province. Our results suggest that dengue infections are increasing in magnitude and expanding out of the lowland areas to higher elevations over time.

**Conclusions:**

There is a high risk of dengue outbreak in the lowland Terai region, with increasing spread towards the mid-mountains and beyond as seen over the last 14 years. Urgent measures are required to increase the availability of diagnostics and resources to mitigate future dengue epidemics.

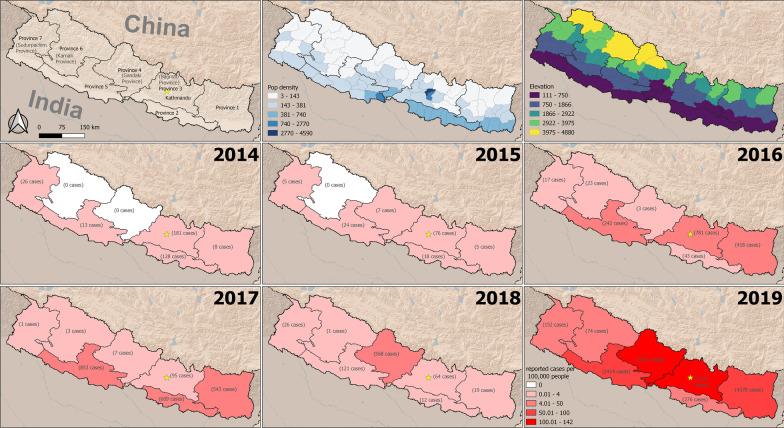

**Supplementary Information:**

The online version contains supplementary material available at 10.1186/s40249-021-00837-0.

## Background

Nepal has seen the outbreak of several emerging and re-emerging diseases in recent years, including dengue fever, rickettsial fevers, and other vector borne diseases [1]. The emergence of these diseases has been attributed to ecological changes, climate change, dispersion of mosquito vectors [2] and human population dynamics [1]. Nepal has three major ecological zones: the tropical Terai region, a subtropical and temperate mid-hill region, and the subalpine to alpine Himalayan region [3, 4].

Dengue fever, malaria, and Japanese encephalitis (JE) are among the most common vector borne diseases (VBDs) in low- and middle-income countries (LMICs) [5]. The endemicity and overall burden of VBDs in LMICs is strongly related to infrastructural weaknesses, including poor water systems, sanitation, and hygiene; and the health system to respond [6]. Studies suggest the co-circulation of similar VBDs like dengue and Japanese encephalitis (JE) for years in Nepal [7]. Recent outbreaks of dengue fever in Nepal in 2019 have alarmed public health authorities with unprecedented spread, morbidity and mortality.

Dengue is a viral infection transmitted by female *Aedes aegypti* and *Aedes albopictus* mosquitoes [8]. The causative agent, dengue virus (DENV), belongs to the genus *Flavivirus* of Flaviviridae family of single-stranded RNA virus [9, 10]. DENV has four main serotypes: DENV-1, DENV-2, DENV-3 and DENV-4 [11]. Infection with any one of these serotypes likely confers lifelong immunity to that specific serotype [12]. Infection by a new serotype may result in severe disease [13]. Most dengue infections (up to 60%) are self-limiting [14], and are characterized by acute fever, frontal headache, vomiting, myalgia, joint pain, and macular skin rash [15]. However, some patients may develop life-threatening conditions such as acute dengue hemorrhagic fever (DHF), dengue shock syndrome (DSS), and (multi-)organ failure [16]. In the absence of effective vaccines and antiviral drugs, symptomatic treatment and vector control programs are currently the only viable strategies for dealing with dengue infections [17, 18]. Studies so far have suggested that timely diagnosis and clinical management with intravenous rehydration are critical to mitigate the severity of infection [19]. Transmission can be reduced through protection from blood feeding *Aedes* mosquitoes.

The laboratory diagnosis of dengue is supported by the clinical suspicion followed by diagnostics that include rapid diagnostic tests (RDT), enzyme linked immunosorbent assay (ELISA) and complete blood counts (CBC) [20]. A CBC profile demonstrating leucopenia, thrombocytopenia, increased hematocrit and liver enzymes are some of the parameters that aid in clinical suspicion [20]. More specific and sensitive diagnostic tools such as viral isolation and culture, and detection of viral genome by polymerase chain reaction (PCR), are not routinely performed in Nepal [20]. Moreover, serological tools are used even during epidemic outbreaks, which further limits the proper diagnosis of disease in Nepal, as such tests are not the gold standard and DENV virus may not be detected prior to the development of antibodies, severely limiting diagnosis during outbreaks [21].

Previous studies from Nepal have explored the seroprevalence in various regions since the first potential outbreak of dengue in Nepal in 2006. Overall seroprevalence of 10.4% (anti-DENV-IgG) was found among suspected cases of dengue fever (DF) and DHF in south-west region of Nepal 2006 [21]. Seroprevalence studies targeting smaller geographic locations have found 7.7% in Kathmandu in 2007 [22], 29.3% in south-western Terai between 2007 and 2008 [23], 9.8% in 2009 in the same region [24], 12.2% in Kanchanpur [25], 11.8% Bharatpur and Rapti Zonal Hospital in 2011 [26, 27], and 19.3% in Chitwan and Dang in 2013[28]. ELISA was the choice of technique in all these studies. Rapid diagnostic tests and particle agglutination tests were used for primary screening. In few studies, molecular techniques such as reverse transcriptase PCR were also used [20, 23]. Despite of these various methods, seroprevalence in the range of 10 to 30% in Nepal.

Although the Government of Nepal has developed an Early Warning and Reporting Systems (EWRS) to issue warning on potential outbreaks, the response to dengue outbreaks have not been sufficient to prevent outbreaks. In 2019 there was a large dengue epidemic in Nepal [29], coinciding with outbreaks of dengue and other *Aedes*-spread diseases throughout much of the tropical world. There are several challenges for prevention and control of dengue infection in Nepal, among which robust mechanism to respond to the outbreak has been constrained by lack of updated epidemiological data. In addition, Nepal has recently entered into a federal system with three tiers of government: federal, provincial and local which lack effective coordination that has adversely impacted the management of human resources, logistic chain management and surveillance [30]. To mitigate these challenges, the federal system has devised an integrated vector control strategy (that includes diseases such as malaria, and kalaazar), that is currently under preparation. Nonetheless, variation in characteristics of vectors, mechanism of disease transmission and epidemiology may remain as major challenges.

Countering these challenges is critical for designing an effective dengue control and prevention program which largely relies on effective detection of the cases, diagnosis and prevention based on the surveillance data. There are no previous studies systematically exploring the epidemiological trends and distribution of the dengue cases at a nationwide scale. The main objective of this study was to explore the epidemiological patterns of dengue fever since its first outbreak (2006) through 2019 in Nepal so that future public health efforts can be appropriately targeted.

## Methods

### Study design

This study is a retrospective analysis of reported dengue case data available from the Epidemiological Disease Control Division (EDCD), under the Ministry of Health and Population, Government of Nepal. Dengue data were extracted from EDCD record. The data presented in this study represents serological diagnosis using rapid test kit [SD Bioline dengue IgG/IgM antibody up to 2015; and after 2015, SD Bioline dengue duo (dengue NS1 Ag + IgG/IgM), (*SD*, *Bio line*, Korea), IgM ELISA was used] of dengue cases. The data in this study covers a period of 14 years (2006–2019)*.*

### Study site

Nepal is a landlocked nation bordering India on the South, East and West; and China on the North. Since the declaration of a new constitution in 2015, Nepal has been divided into seven provinces [Province-1, Province-2, Bagmati Province (Province 3), Gandaki Province (Province 4), Province-5, Karnali Province (Province 6) and Sudurpaschim Province (Province 7)] and 77 districts with area of 147,516 km^2^. It occupies 0.3% of the land region in Asia and 0.03% in the world. Nepal is located between 26° 22′ N to 30° 27′ N and longitude 80° 4′ E to 88° 12′ E. The general landscape of Nepal includes the lowland swamp Terai region at 70 m from ocean level to the highest elevation in the world: Mount Everest (8848 m). Land divisions incorporate Terai, Hills and Mountains. The most recent statistics in 2011 estimated a population of 26.5 million with a development pace of 1.35 individual per annum [31]. Over half of the population lives in the Terai district of Nepal, where vector borne diseases such as malaria, dengue, Japanese encephalitis, visceral leishmaniasis (kala-azar) fever are endemic.

### Data collection

Data on dengue surveillance is collected by the health system infrastructure that includes Health Posts, Primary Health Centers (PHC), District Hospitals, Provincial Hospitals and Central Hospitals. Dengue cases recorded in the health center are collected monthly and are reported to the District Health Office (DHO)/District Public Health Office (DPHO). The information is subsequently reported to the Epidemiology and Diseases Control Division (EDCD) from DHO/DPHO on a monthly basis through the Health Management Information System (HMIS)-reporting mechanism. Besides HMIS, an Early Warning Reporting System (EWARS) is also utilized to record hospital admitted dengue cases and dengue deaths. Population density at the district level was calculated as people per km^2^. District level population counts were derived from the 2011 Nepal Census. We calculated mean elevation for each district using elevation data from the GTOPO30 global digital elevation model (DEM).

### Data analysis

Data were first entered in Microsoft Excel 2010 (Microsoft, Seattle, WA, USA) for analysis. Trends in incidence of reported dengue cases and proportions of dengue cases in different province/districts in Nepal (2006–2019) were analyzed. Districts level of dengue cases were available for 2016 through 2019. A map of dengue cases was created for 2016 through 2019. A mixed effects negative binomial regression was used to test for associations between reported case incidence and calendar year, mean elevation at the district level, and district population density. A random intercept was used for district to account for repeated observations within each district across the calendar years. The outcome variable was reported case incidence per 100 000 per year, rounded to the nearest whole number. We hypothesized that incidence was increasing at higher elevations over time and included an interaction term between calendar year and mean elevation to test our hypothesis. Both population density and mean elevation were centered on their mean values and standardized using their respective standard deviations so that a one-unit change in both values corresponds to one standard deviation change.

All maps and map layers were created using QGIS version 3.4 (https://qgis.org/en/site/). The negative binomial regressions were done using R statistical software version 3.5.2.

## Results

### Annual trend of dengue incidence in Nepal (2006–2019)

**Fig. 1:**
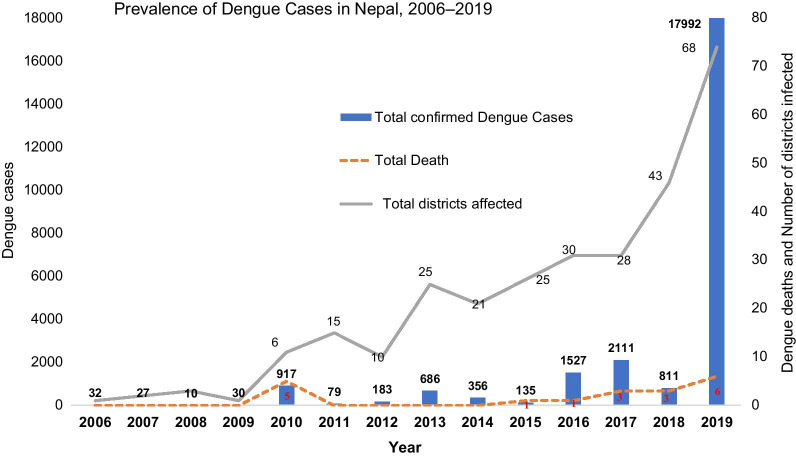
Prevalence of dengue in Nepal: 2006–2019

The trend of dengue (confirmed by serological test either IgM ELISA or rapid test kit) incidence over the period of 2006–2016 was analyzed (Fig. 1). The trends of dengue incidence are presented below in different intervals, ranging from 1 to 4 years.

### Dengue in 2006

Nepal reported its first dengue case in a Japanese foreigner, imported from India in 2004. Two years later there was an endogenous outbreak in lowland Chitwan district in 2006; with a total of 32 reported dengue cases throughout the country.

### Period between 2007 and 2010

From 2007 to 2009, the total number of reported dengue cases was slightly less in comparison to the 2006 outbreak. In 2007, 27 dengue cases were reported from four districts of Terai region. However, there was slight decrease in number of dengue cases; reported 10 from three districts. In 2010 Nepal faced a major outbreak of dengue, with 917 reported cases and 5 reported deaths (2 from Chitwan district, 1 from Nawalparasi and 2 from Rupandehi district) from six districts of Nepal (Fig. 1).

### Period between 2011 and 2013

In 2011, the number of dengue cases (79 cases) were very low in comparison to 2010. However, there was an expansion in its distribution: cases were reported from 15 districts of Nepal (only 6 districts in 2010 epidemic). There was another dengue epidemic in 2013 and a total of 686 cases were reported from 25 districts of Nepal. There were no reports of DENV-related deaths between 2011 and 2013.

### Period between 2014 and 2016

**Fig. 2:**
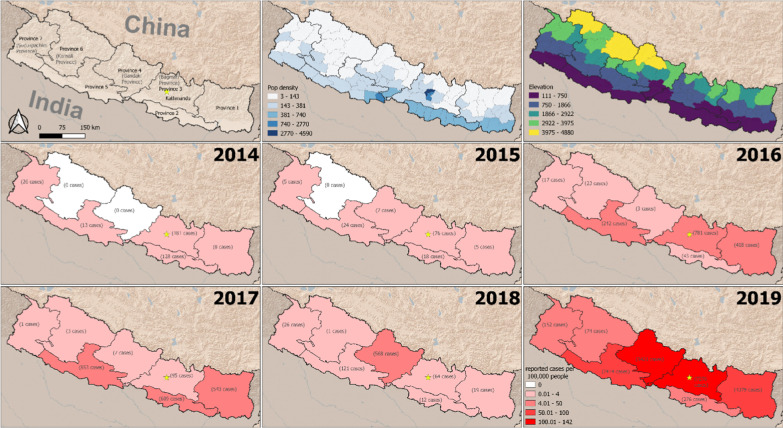
Province wise dengue cases in Nepal, 2014–2019

In 2014, a total of 356 dengue cases were reported from 21 districts of Nepal. Out of the 356 cases, 50.8% (181/356) were from Bagmati Province, 35.9% (128/356) from Province-2, 7.3% (26/356) from Sudurpaschim Province, 3.6% (13/356) from Province-5 and 2.2% (8/356) from Province-1. There were no dengue cases reported from Karnali Province and Gandaki Province in 2014 (Fig. 2). In 2015, there were only 135 reported dengue cases throughout the country, most from Bagmati Province (76 cases out of 135 cases) and 1 death from Dang District. In 2016, there was another dengue epidemic in Nepal and a total of 1527 dengue cases were reported from 30 districts; comprising all seven Provinces. Only one dengue death was reported from Chitwan District in 2016 epidemic. Province-wise dengue cases from 2016 showed 51.2% (781/1527) from Bagmati Province, 27.4% (418/1527) from Province -1, 15.8% (242/1527) from Province-5, 2.8% (43/1527) from Province-2, 1.5% (23/1527) from Karnali Province, 1.1% (17/1527) from Sudurpaschim Province; and only three dengue cases were reported from Gandaki Province (Fig. 2). Among 1527 cases, 44.8% (687/1527) were from Chitwan district (Bagmati Province) and 26.5% (405/1527) were from Jhapa District (Province 1) in 2016 (Additional file 1: Table S1).

### Period between 2017 and 2019

In 2017, a total of 2111 dengue cases were reported from 28 districts of Nepal. The number of cases rose by 38% (1527 in 2016 versus 2111 in 2017) in comparison to 2016. Out of 2111 cases, 40.4% (853/2111) were from Province-5, 28.8% (609/2111) from Province-2, 25.7% (543/2111) from Province-1, and 4.5% (95/2111) from Bagmati Province. There were three dengue deaths reported each from Palpa, Chitwan and Makawanpur districts in 2017. In 2018, there were only 811 reported cases throughout the country, most from Gandaki Province (568 cases out of 811 cases). There was a geographic expansion in reported cases, now from 43 districts of Nepal. There were three deaths reported in Rupandehi (two cases) and Makawanpur district (1 case).

In 2019, there was a large dengue epidemic in Nepal, with a total of 17 992 reported dengue cases from 68 districts; comprising all seven Provinces. There were six dengue deaths reported from five districts of Nepal (2 deaths in Chitwan, and one each death in Sunsari, Sindhupalanchock, Kathmandu and Doti) in the 2019 epidemic. Province-wise dengue cases distribution in 2019, 40.5% (7276/17 992) were from Bagmati Province, 24.4% (4379/17 992) from Province-1, 19% (3421/17 992) from Gandaki Province, 13.4% (2414/17 992) from Province-5, 1.5% (276/17 992) from Province-2, 0.8% (152/17 992) from Sudurpaschim Province and very low (0.4%; 74/17 992) from Karnali Province (Fig. 2). On district wise distribution, Sunsari District comprised 19% (3431/17 992) followed by Chitwan (18.9%; 3402/17 992), Kaski (15.7%; 2824/17 992), Kathmandu (8.8%; 1589/17 992), Lalitpur (3.3%; 596/17 992) and Jhapa (2.9%; 525/17 992) (Additional file 1: Table S1).

### Spatial distribution and ecological analysis of reported dengue fever case incidence at the district level (2016–2019)

**Fig. 3:**
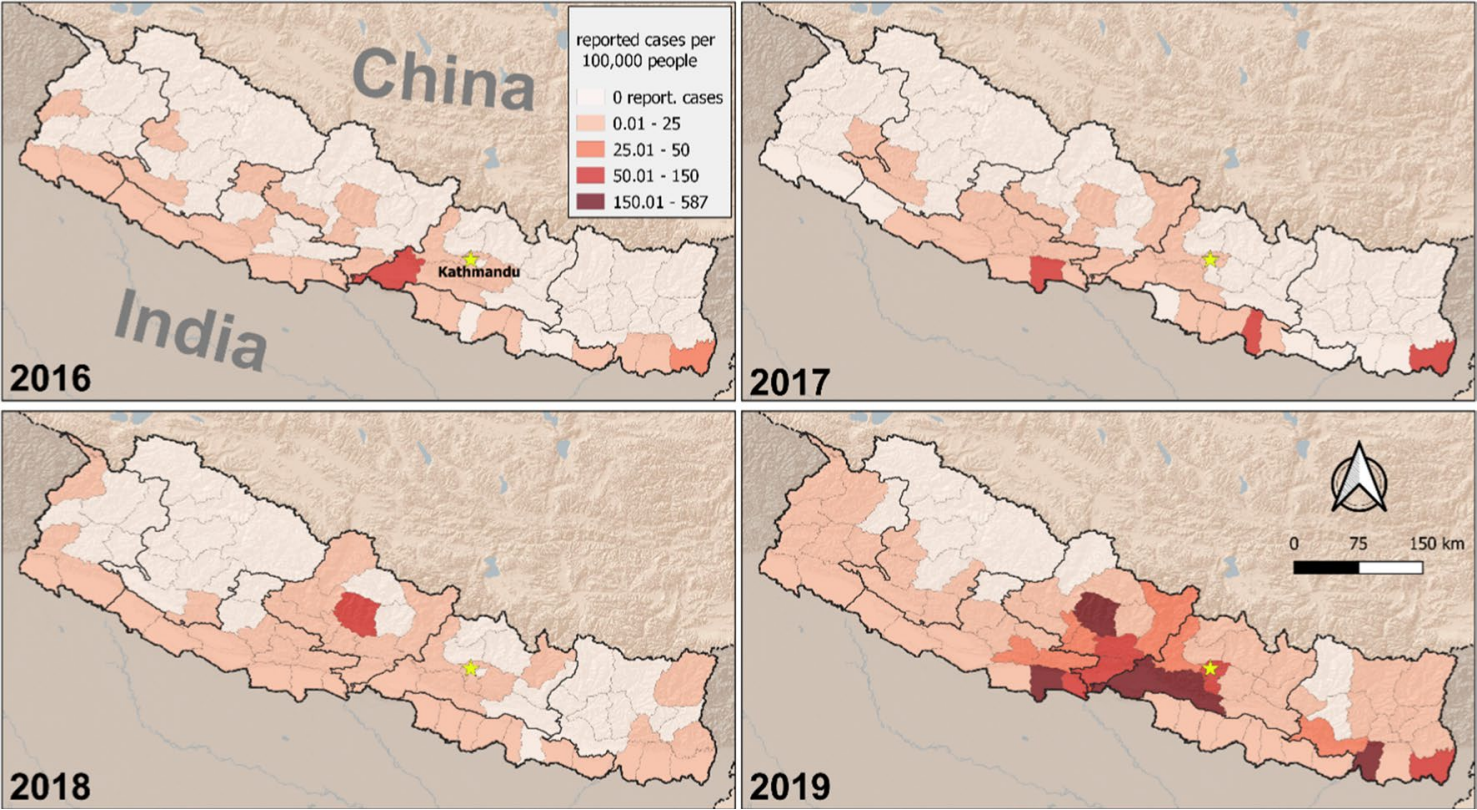
Choropleth maps of reported dengue fever case incidence at the district level (2016–2019). Case incidence is presented as number of cases per 100 000 people for each year

Choropleth maps of reported dengue fever case incidence at the district level (2016–2019) were generated, with case incidence presented as the number of cases per 100 000 people for each year (Fig. 3).

Reported case incidence was much higher in 2018 and 2019, using 2016 as a comparison (Additional file 2: Table S2). The incidence was approximately five times higher in 2018 (incidence rate ratio (IRR): 4.8; 95% confidence interval (*CI*) 1.5–15.3) and over 140 times higher in 2019 (IRR: 141.6; 95% *CI* 45.8–438.4). Population density was not a statistically significant predictor of case incidence. Mean elevation had a negative association with case incidence (Table 1). A one standard deviation increase in elevation was associated with a 90% decrease in reported case incidence (IRR: 0.10; 95% *CI* 0.01–0.20). However, the association with mean elevation varied across the years, as is evident from the interaction effect in our model. In comparison to 2016, incidence was greater at higher elevations in 2018 (IRR: 22.7; 95% *CI* 6.0–86.1) and 2019 (IRR: 9.6; 95% *CI* 2.6–36.1).

**Table 1:** Results from the mixed effects negative binomial regression for predictors of reported dengue fever case incidence at the district level

Covariate	IRR (95% *CI*s)
Year	
2016	Comparator
2017	3.1 (0.9–10.8)
2018	4.8 (1.5–15.3)
2019	141.6 (45.8–438.4)
Mean elevation	0.1 (0.01–0.2)
Population density (people per km^2^)	1.3 (0.9–1.8)
Year and elevation interaction	
2016 × mean elevation	Comparator
2017 × mean elevation	1.8 (0.4–7.6)
2018 × mean elevation	22.7 (6.0–86.1)
2019 × mean elevation	9.6 (2.6–36.1)

*IRR* Incidence rate ratio, *CI* Confidence interval

## Discussion

Since the first report of dengue fever in 2004 [32], Nepal has steadily experienced a rise and expansion of cases, with 17 992 cases in 2018/2019 from most districts (68 out of 77 districts). The choropleth maps (2016–2019) of reported dengue fever case incidence at the district level showed dengue incidence was five times higher in 2018 and over 140 times higher in 2019. Such a steady rise and nationwide distribution of dengue makes the disease a national priority with urgent implications for control and prevention.

### Trends of dengue cases in Nepal

The overall trend of dengue incidence and its distribution show a rising trend with outbreaks in 2010, 2013, 2016 and 2019 in Nepal. In just last 6 years since the first imported case of dengue in 2004, Nepal has become an endemic country for dengue. Following the outbreak of DF/DHF in India in 2006, a minor outbreak was confirmed in the same year in Nepal [21, 23] with 32 cases but no fatalities. All four serotypes of DENV were isolated in the 2006 outbreak from nine districts of lowland tropical Terai whereas the populous hilly districts including Kathmandu and Pokhara were spared from the outbreak [20]. The primary vector of dengue transmission, *Ae. aegypti,* was reported from only five districts bordering India implying a possibility of high importation of cases [20]. A previous study also showed clustering of dengue cases in border districts with India and were attributed to favorable climatic conditions, high population density, and high population movement across the border [33].

Dengue remained almost latent during the period of 2007 and 2009, until the massive outbreak of 2010 with 917 cases and distribution into six districts. One study conducted in southern Terai during this period showed a high prevalence (29.3%) of anti-DENV IgM [23]. Subsequent studies suggested lower seroprevalence, one showing a seroprevalence of 9.8% in 2009 [24] and an extensive cross-sectional study covering southern Terai showing an overall seroprevalence of 12.1% with a high proportion in Kanchanpur bordering India [25]. These studies showed the high vulnerability and impending epidemic outbreak in the Terai region [25]. Around 80% of the total confirmed cases were reported from Terai region that showed all serotypes with entomological evidence of both vectors: *Ae. aegypti* and *Ae. albopictus* [21].

The outbreak of 2016 was the result of re-emergence of DENV-1 that recorded 1527 cases, with distribution in 30 districts of Nepal. Two terai districts: Chitwan and Jhapa accounted almost 72% (1092/1527) of all reported cases. Both of these districts have a tropical climate, and border with India which can explain in part the high incidence of dengue cases [34]. Following the first report of dengue in the highland region in 2010, 3.1% of the total cases, with 0.4% from Kathmandu alone were reported by the end of 2016. The National Public Health Laboratory (NPHL) Kathmandu reported 16.9% (45 out of 266) of patients showing anti dengue IgM antibodies in serum [35].

Triennial peaks and the expansion in distribution of dengue epidemics in 2010, 2013, 2016 and 2019 are in line with the previous reports from Brazil [36] and Cuba [37]. In the subsequent outbreaks, serious complications associated with the dengue infection were not observed as expected and could be due to prevalence of a newer serotype (DENV-2) in the 2013 outbreak [38]. This could be due to the low virulence of newer strain, or cross-immunity developed due to endogenous infection. Also, the higher mortality and morbidity are associated with secondary infection with another serotype. Antibody dependent enhancement has been shown to be causing severe form of dengue, also known as secondary explosion as is observed in India, Bangladesh [39]; Vietnam, Singapore and Senegal [40]. The same mechanism may have a role in the outbreak that occurred in 2016 and 2019 in Nepal; and poses risk for future outbreaks.

### Geospatial distribution of dengue cases

In 2010, DENV-1 was the prominent serotype for the epidemic. However, the outbreak of 2013 was solely caused by the DENV-2 [38, 41]. This indicated the prevalence of all serotypes with endemicity of DENV as silent threat all over the country. Three lowland Terai districts–Chitwan, Jhapa, and Parsa were again worst hit districts, together constituting 85% of all reported cases in the country. Over the years, the rising incidence of dengue in Kathmandu has countered the presumption that Kathmandu was climatically unsuitable for dengue vectors. Increased urbanization, industrialization together with the climate change may have contributed a conducive ambient environment for *Aedes* vector mosquitoes [42–44].

The outbreak of 2016 showed both an increase in the number of cases and the distribution of disease to newer temperate zones within Nepal. For instance, temperate hilly zones of Gandaki province began to report cases in 2015 while the outbreak in subsequent year affected Karnali province located in upper hilly region. Also, the outbreak of 2016 marked the geographic expansion of dengue infections in all seven provinces. The emergence and re-emergence of DENV serotypes intermittently in varying manifestations implies the possible burst of severe forms of dengue-related illnesses. Similar mechanism and patterns of DENV infection with multiple virus clades were observed in Indonesia [45] and Brazil [46] while circulation of DENV-1 in the same period (2014–2016) was also observed in China [47] and other South Asian countries: India [48], Bangladesh [49], Pakistan [50] and Sri Lanka [51]. In our study, dengue case incidence showed five times higher incidence in 2018 and over 140 times in 2019 in comparison to 2016 (Table 1). The findings of our study are in line with studies reported from Thailand [52] and Bangladesh [53].

Dengue poses a serious public health threat and economic challenge globally and in Nepal. Multipronged vector control strategies that are cost effective, sustainable and environmentally friendly are gaining increasing priority. Currently, newer vector control methods such as sterile insect technique, production of genetically modified vectors and paratransgenesis are being studied in various parts of the world. Also, innovative vaccine candidates have been used for the prevention of dengue infections. The use of tetravalent dengue vaccine (CY-D-TDV) has been found to be effective for the treatment of dengue infections [54]. Nonetheless, there are various constraints and urges the need for a multi-pronged approach including vaccine development [44, 55].

### Impact of climate change and ecology

There was a steady rise in number of cases and its distribution between the period of 2017 and 2019. The outbreak is remarkable for its spatial and temporal shift in addition to the role of two serotypes (DENV-1 and DENV-2) [1]. Kathmandu saw the repeated outbreak of dengue and since then experts fear the imminent outbreak of dengue in future. Although the vectors are thought to normally only fly 500 m in their lifetime [56, 57], a number of underlying factors such as urbanization, trade and transit from dengue-infested regions and climate change are favoring their spread and potency. Specifically, changes in temperature and rainfall in upland hilly regions and relative humidity in lowland plains are established as contributing factors for rise and distribution of vectors [58].

The primary vectors: *Aedes aegypti* and *Aedes albopictus—*depend upon temperature and precipitation for their growth, survival and feeding behavior [59] and also affects the vector-human transmission cycle [60]. Increasing temperature in the region can provide a favorable environment for dengue vectors and its transmission. The latest dengue outbreak of 2019 may have been flared up by unexpected early rains which may have accelerated the outbreak as early as on May 13, 2019 from Sunsari district [29]. Similarly, annual monsoon season of each year in the country makes ambient room for mosquitoes by its high humidity while the post-monsoon period favors their breeding and transmission by high rainfall and heavy flooding [1]. Some prevailing findings have suggested the existence of dengue vectors (*Aedes aegypti* and *Aedes albopictus*) from the tropical lowland to the highland Dhunche, Rasuwa (2100 m elevation) district in Nepal [61]. This geographical expansion of dengue fever is likely the result of vector habitat expansion, which may be a result of global warming [58].

### Implications for national dengue control program

The government of Nepal has released the national guidelines for the prevention, control, and management of dengue in the country which has focused on vector-control strategies as the best policy to curb epidemics. Despite the guideline, the rising trend of dengue cases and expansion in geographic distribution in almost all the districts of Nepal poses significant challenges. Of the challenges, Nepal can plan through the historical account of dengue epidemiology, rising trend and its spread in the districts. Specifically, the visualization of dengue cases among the districts can also help in categorizing and prioritizing the districts based on the epidemiological burden identified in this study. Also, the dengue control and prevention program can incorporate spatially focused strategies to ensure the preventive measures such as distributing mosquito repellants, clearing of puddles (or water collection around the households), bushy areas, and fumigation. Targeted programs with allocation of resources for treatment and prevention can be planned based on the spatio-temporal distribution of the cases visualized in this study. In addition, integrated vector control programs may benefit from the comprehensive data of dengue cases and its distribution for resource allocation.

## Strengths and limitations

This study has consolidated the national dengue data since the first report of dengue in Nepal up to the present. In addition to integrating all the data through epidemiological analysis, this study reveals trends in space and time which can inform the dengue control and prevention program of Nepal. This study has several limitations. Due to resemblance with other symptoms of tropical diseases, dengue cases may have been undetected and overlooked, posing a challenge on reporting [53]. In our study, the majority of dengue cases were diagnosed by ELISA (IgM/IgG antibody detection) which may have failed to detect dengue virial infection in the early stages and can give false positive result in a patient who had past dengue infections or any other infections by flaviviruses. The study relied on the retrospective data from government’s EDCD which may have missed private sector data and thus may not reflect the true extent of the dengue burden in Nepal. During the outbreaks due to logistic shortages, reporting of the cases were not uniformly confirmed by RDTs, sometimes were based on the clinical diagnosis.

## Conclusions

Nepal is experiencing a major increase the burden of dengue fever*.* While cases were once limited to the tropical lowland Terai region, they now occur at higher elevations and with increasing case loads. Chikungunya and Zika viral infections are both spread by the same vectors, *Aedes aegypti* and *Aedes albopictus*. Therefore, there will be chance of spread of these infections since dengue has become endemic. Urgent measures are required to increase the diagnostics and resources to mitigate the epidemic burden of dengue in Terai and peripheral regions. Findings from this study can inform the national dengue control and prevention program in resource allocation and priority setting with implications for future epidemics.

## Supplementary Information

**Additional file 1: Table S1.** District wise cases of dengue in 2016, 2017, 2018 and 2019 in Nepal.

**Additional file 2: Table S2.** Negative binomial regression for predictors of dengue fever case incidence at the district level, stratified by year.

## Data Availability

All data pertaining to this study are within the manuscript.

## Abbreviations

EDCD: Epidemiology Diseases Control Division
IRR: Incidence rate ratio
VBDs: Vector borne diseases
LMICs: Low- and middle-income countries
JE: Japanese encephalitis
DENV: Dengue virus
DF: Dengue fever
DHF: Dengue hemorrhagic fever
DSS: Dengue shock syndrome
RDT: Rapid diagnostic tests
ELISA: Enzyme linked immunosorbent assay
CBC: Complete blood counts
PCR: Polymerase chain reaction
EWRS: Early Warning and Reporting Systems
DHO: District Health Office
DPHO: District Public Health Office
DEM: Digital elevation model
NPHL: National Public Health Laboratory
*CI*: Confidence interval
