# Supplementary material for: Epidemiology of dengue virus infections in Nepal, 2006–2019
Source: Infect Dis Poverty. 2021 Apr 15;10:52. doi: 10.1186/s40249-021-00837-0 (PMC8047528; doi:10.1186/s40249-021-00837-0)
Supplement: Supplementary file 1 — Additional file 1: Table S1. District wise cases of dengue in 2016, 2017, 2018 and 2019 in Nepal. [file 40249_2021_837_MOESM1_ESM.docx]

Additional file 1: Table S1: District wise cases of dengue in 2016, 2017, 2018 and 2019 in Nepal

| **District** | **2016** | **2017** | **2018** | **2019** |
| --- | --- | --- | --- | --- |
| Jhapa | 405 | 543 | 5 | 525 |
| Morang | 9 | 0 | 2 | 183 |
| Sunsari | 4 | 0 | 8 | 3431 |
| Bhojpur | 0 | 0 | 0 | 17 |
| Udaypur | 0 | 0 | 0 | 134 |
| Illam |  |  | 1 | 25 |
| Taplejung |  |  | 1 | 4 |
| Dhankuta |  |  | 2 | 5 |
| Sankuwasaba |  |  |  | 10 |
| Pachthar |  |  |  | 33 |
| Terathum |  |  |  | 5 |
| Okhaldhunga |  |  |  | 7 |
| Province-1 | **418** | 543 | 19 | 4379 |
| Saptari | 1 | 0 | 2 | 28 |
| Siraha | 0 | 0 | 1 | 19 |
| Dhanusa | 0 | 27 |  | 7 |
| Mahottari | 3 | 438 | 3 | 10 |
| Sarlahi | 28 | 130 | 2 | 49 |
| Bara | 4 | 2 | 1 | 60 |
| Parsa | 7 | 0 | 2 | 71 |
| Rautahat | 0 | 12 | 1 | 32 |
| province-2 | 43 | 609 | 12 | 276 |
| Kavre | 2 | 0 | 1 | 53 |
| Lalitpur | 1 | 0 | 1 | 596 |
| Bhaktapur | 0 | 1 | 0 | 364 |
| Kathmandu | 5 | 1 | 16 | 1589 |
| Dhading | 4 | 67 | 7 | 168 |
| Makwanpur | 82 | 3 | 9 | 961 |
| Chitwan | 687 | 23 | 28 | 3401 |
| Nuwakot |  |  |  | 49 |
| Sindhuli |  |  |  | 29 |
| Dolakha |  |  | 2 | 22 |
| Rasuwa |  |  |  | 3 |
| Ramechap |  |  |  | 24 |
| Sindhupalchok |  |  |  | 17 |
| Provience-3 | 781 | 95 | 64 | 7276 |
| Gorkha | 0 | 1 | 2 | 78 |
| Syangja | 1 | 1 | 4 | 114 |
| Kaski | 1 | 1 | 553 | 2824 |
| Baglung | 1 | 4 | 4 | 39 |
| Tanahu | 0 | 0 | 1 | 271 |
| Parbat |  |  | 2 | 44 |
| Mustang |  |  | 1 | 0 |
| Myagdi |  |  | 1 | 24 |
| Manang |  |  |  | 1 |
| Lamjung |  |  |  | 26 |
| province-4 | 3 | 7 | 568 | 3421 |
| Argakhachi | 0 | 21 | 4 | 56 |
| Palpa | 0 | 14 | 7 | 70 |
| Nawalparasi | 3 | 37 | 15 | 507 |
| Rupandehi | 164 | 677 | 61 | 1476 |
| Kapilbastu | 3 | 57 | 8 | 139 |
| Pyuthan | 1 | 12 | 3 | 19 |
| Rolpa | 0 | 4 |  | 23 |
| Rukum | 1 | 0 |  | 0 |
| Dang | 8 | 13 | 2 | 2 |
| Banke | 37 | 1 | 6 | 43 |
| Bardiya | 25 | 0 | 4 | 32 |
| Gulmi | 0 | 17 | 10 | 47 |
| Province-5 | 242 | 853 | 121 | 2414 |
| Surkhet | 22 | 2 | 0 | 47 |
| Kalikot | 1 | 0 | 0 | 5 |
| Dailekh | 0 | 1 | 0 | 9 |
| West Rukum |  |  |  | 4 |
| Salyan |  |  | 1 | 9 |
| Karnali Pardesh-6 | 23 | 3 | 1 | 74 |
| Doti | 0 | 0 |  | 6 |
| Kailali | 14 | 0 | 3 | 56 |
| Kanchanpur | 2 | 0 | 13 | 31 |
| Dadeldhura | 0 | 0 | 2 | 13 |
| Acham | 0 | 1 |  | 8 |
| Baitadi | 1 | 0 |  | 9 |
| Darchula |  |  | 9 | 25 |
| Bajhang |  |  |  | 4 |
| Province-7 | 17 | 1 | 27 | 152 |
| **National** | **1527** | **2111** | 811 | 17992 |
