# Supplementary material for: Epidemiology of dengue virus infections in Nepal, 2006–2019
Source: Infect Dis Poverty. 2021 Apr 15;10:52. doi: 10.1186/s40249-021-00837-0 (PMC8047528; doi:10.1186/s40249-021-00837-0)
Supplement: Supplementary file 2 — Additional file 2: Table S2. Negative binomial regression for predictors of dengue fever case incidence at the district level, stratified by year. [file 40249_2021_837_MOESM2_ESM.docx]

**Additional file 2: Table S2**: Negative binomial regression for predictors of dengue fever case incidence at the district level, stratified by year.

|  | **IRR (95% CIs)** | | | |
| --- | --- | --- | --- | --- |
| **covariate** | **2016** | **2017** | **2018** | **2019** |
| mean elevation | 0.01 (0.00 - 0.13) | 0.06 (0.02 - 0.21) | 1.92 (1.06 - 3.48) | 0.59 (0.41 - 0.86) |
| population density (people per km2) | 0.09 (0.01 - 1.49) | 0.61 (0.21 - 1.78) | 1.05 (0.58 - 1.90) | 1.44 (1.00 - 2.09) |
